# Preimplantation genetic testing for complex chromosomal rearrangements: clinical outcomes and potential risk factors

**DOI:** 10.3389/fgene.2024.1401549

**Published:** 2024-07-29

**Authors:** Dun Liu, Chuangqi Chen, Qianwen Huang, Yunqiao Dong, Liqing Xu, Mei Dong, Zhenghong Zhu, Li Huang, Fang Wang, Lijia Zhang, Xiqian Zhang, Fenghua Liu

**Affiliations:** Reproductive Medical Center, Guangdong Women and Children Hospital, Guangzhou, Guangdong, China

**Keywords:** complex chromosomal rearrangements (CCR), PGT, NGS, carrier’s sex, genetic counseling

## Abstract

**Objective:**

Complex chromosome rearrangements (CCR) are rare structural abnormalities involving at least three breakpoints, categorized into three types based on their structure: type A (three-way rearrangements), type B (double two-way translocations), and type C (exceptional CCR). However, thus far, limited data exists on preimplantation genetic testing for chromosomal structural rearrangements (PGT-SR) in CCR carriers. This study aims to evaluate the clinical outcomes and influencing factors of PGT-SR in couples with CCR.

**Methods:**

Fifteen couples with unique CCR recruited from 793 couples following PGT-SR between January 2017 and May 2023. In addition, a total of 54 CCR cases, 39 previously reported as well as 15 newly added, were included in the analysis of factors associate with normal/balanced embryos.

**Results:**

A total of 100 blastocysts were biopsied and analyzed in 15 CCR couples after 17 PGT-SR cycles, with 16.0% being euploid, 78.0% aneuploid and 6.0% mosaic. 11 normal/balanced embryos and one mosaic embryo were transferred, resulting in eight live births. Furthermore, based on the combined data from 54 CCR carriers, the proportion of normal/balanced embryos was 10.8%, with a significant decrease observed among female carriers compared to male heterozygotes (6.5% vs. 15.5%, *p* = 0.002). Type B exhibited the lowest rate of euploid embryos at only 6.7%, followed by type A at 11.6% and type C at 14.0%, although the differences were not significant (*p* = 0.182). After completing the multivariate generalized estimating equation (GEE) analysis, type B (*p* = 0.014) and female carrier (*p* = 0.002) were identified as independent risk factors for fewer euploid embryos.

**Conclusion:**

The occurrence of balanced CCR in patients with reproductive abnormalities may be more frequent than we expected. Despite the proportion of normal/balanced embryos being significantly low, which can be influenced by CCR type and carrier’s sex, PGT-SR may improve the reproductive outcomes among CCR cases. These findings can optimize the clinical management and genetic counseling of CCR carriers seeking assisted reproductive technology (ART).

## Introduction

Complex chromosomal rearrangements (CCR) are structural abnormalities characterized by two or more chromosomes with at least three breakpoints, resulting in the exchange of genetic material between non-homologous chromosomes (Pellestor et al., 2011a). CCR can be classified into three categories according to their structure (Kausch et al., 1988; Pellestor et al., 2011a): type A (three-way rearrangements), type B (double two-way translocations) and type C (exceptional CCR) respectively; the first two types involve only translocations, while exceptional CCR often combine translocations with other structural aberrations such as inversions and insertions. CCR occurring in phenotypically normal persons are extremely rare, either familial or *de novo*, with an estimated incidence of 0.003% in newborns (Giardino et al., 2009). Nevertheless, they are more prevalent in individuals with reproductive abnormalities such as infertility, recurrent pregnancy loss and/or offspring abnormality, ranging from 0.1% to 0.2% frequency (Mau- and Holzmann, 2005; Liao et al., 2017). For carriers of balanced CCR, it is likely that a significant number of unbalanced gametes will be generated due to abnormal segregation patterns during meiosis (Loup et al., 2010; Pellestor et al., 2011b; Godo et al., 2013; Rossi et al., 2023).

Preimplantation genetic testing for chromosomal structural rearrangements (PGT-SR) has been widely applied for carriers of common chromosomal rearrangements, including reciprocal translocations, Robertsonian translocations and inversions (Coonen et al., 2020; Spinella et al., 2023). However, the available data on preimplantation genetic testing for structural rearrangements (PGT-SR) in carriers with CCR remains insufficient. To date, only a few case reports or small series have been published regarding carriers with balanced CCR undergoing PGT-SR (Escudero et al., 2008; Lim et al., 2008; Vanneste et al., 2011; Scriven et al., 2014; Frumkin et al., 2017; Brunet et al., 2018; Hu et al., 2018; Mas et al., 2018; Li et al., 2020; Ou et al., 2020; Dufton and Bouzayen, 2021; Özer et al., 2022; Ren et al., 2023). Here, we assessed the clinical outcomes of 15 couples with novel CCR undergoing PGT-SR using next-generation sequencing (NGS). To our knowledge, this is the most extensive series of CCR carriers undergoing PGT-SR ever reported. Moreover, we conducted a systematic analysis to identify factors influencing the outcomes of PGT-SR in individuals with CCR by integrating our data with previously reported cases. These data would provide valuable insights for the clinical management and genetic counseling of CCR carriers seeking assisted reproductive technology (ART).

## Materials and methods

### Study patients

Fifteen couples were retrospectively selected from a large cohort of 793 couples who underwent PGT-SR at the Reproductive Medicine Center of Guangdong Women and Children Hospital between January 2017 and May 2023. One partner in each couple was a CCR carrier, including five female carriers and ten male heterozygotes. Among the remaining 778 couples, one partner carried a common chromosomal rearrangement, such as reciprocal translocation, Robertsonian translocation or inversion. Classical pericentric inversion “inv (9)(p11q12)” was excluded. The present study was reviewed and approved by the Institutional Review Board (IRB) of Guangdong Women and Children Hospital, ensuring compliance with ethical guidelines. Written consent was obtained from all participating patients prior to their inclusion in the study.

For ease of reference, we assigned numbers to the 15 couples as cases 1 to 15 (Table 1). More than half of the female participants (cases 3, 6, 7, 9, 10, 11, 12, 14 and 15) had experienced abnormal pregnancy outcomes, including spontaneous or induced abortions. Oligoasthenoteratozoospermia (OAT) was diagnosed in three of the male carriers (cases 4, 5 and 11), while the rest exhibited normal sperm parameters. None of these couples had achieved a healthy live birth before undergoing PGT-SR.

**TABLE 1:** Clinical outcomes of PGT-SR cycles for 15 CCR carriers.

					Oocytes				Embryos					
Case	Karyotype of CCR case	Type of CCR	Female age (years)	PGT-SR cycle	Collected	MII	2PN	Day3	Biopsied	Euploid	Aneuploid	Mosaic	Transferred	Pregnancy outcome
1	46, XX, t (2; 9;22) (p25.2; q32; q11.1)	A	35	1	14	12	12	12	3	1	2	0	1	Negative
2	46,XY,t (5; 14; 11) (q23; q24.3; q21)	A	27	1	38	35	23	20	16	4	11	1	2	1 live birth
3	46,XY,t (1; 14) (q32.3; q24.3),t (2; 9) (p11.2; p24.2)	B	33	1	25	19	15	15	2	0	2	0	0	
4	46,XY,t (3; 4) (q21; p15.2)t (4; 22) (p16.1; q12.2)	B	25	1	32	29	23	21	9	0	9	0	0	
5	45,XY,t (3; 13) (q26.2; q21.3),der (13; 14) (q10; q10)	B	29	1	33	30	22	22	12	2	9	1	1	1 live birth
6	46,XX,t (1; 7) (p13.1; q11.23),t (5; 6) (q15; q23)	B	27	1	17	17	16	16	6	0	5	1	0	
7	46,XY,t (4; 5) (q27; q31),t (6; 15) (q27; q25)	B	27	1	40	35	27	26	11	2	9	0	1	1 live birth
8	46,XY,t (7; 22) (q36; q13.1)inv (22) (q12.2q13.1)	C	30	1	15	15	12	12	7	1	6	0	1	Negative
9	46,XX,t (7; 8) (p13; q11.23)inv (7) (p13q21.2)	C	37	1	12	9	7	7	3	1	2	0	1	Negative
				2	14	13	11	11	6	1	5	0	1	1 live birth
10	46,XX,t (2; 18) (p10; p10),inv (9) (q21.2q22.3)	C	24	1	11	11	7	7	5	0	5	0	0	
				2	10	8	6	6	3	0	2	1	1^a^	1 live birth
11	46,XX,der (4)ins (4; 14) (q33; q22q24.2)t (4; 21) (q34; q22.1),der (14)ins (14; 21) (q11.2; q11.2q21)ins (21; 14) (q22.1; q11.2q21)ins (4; 14),der (21)ins (14; 21)ins (21; 14)t (4; 21)	C	30	1	13	11	10	10	4	0	4	0	0	
12	46,XY,der (4)inv (4) (q32q35)t (4; 11) (q32; q25),der (11)t (4; 11)	C	26	1	22	19	15	15	5	1	4	0	1	1 live birth
13	46,XY,der (2)t (2; 10) (q35; q24.3),der (10)inv (10) (q22.1q24.3)t (2; 10)	C	25	1	12	12	10	10	8	3	4	1	2	2 live births
14	46,XY,der (5)ins (7; 5) (p22; p15.1p15.3)t (5; 6) (p15.1; q23),der (6)ins (7; 6) (p22; q22q23)t (5; 6),der (7)ins (7:5)ins (7:6)	C	28	1	20	20	13	13	0	0	0	0	0	
15	46,XY,der (3)t (3; 9) (p22; q34.1),der (9)inv (9) (q33q34.3)t (3; 9)	C	37	1	9	5	1	1	0	0	0	0	0	
Total				17	337	300	230	224	100	16	79	5	12	8 live births

^a^ Mosaic embryo.

Considering the limited prevalence of CCR carriers, we expanded our sample size by collecting data from PGT-SR studies involving individuals with CCR that have been reported in PubMed up until now, with the aim of exploring factors influencing euploidy of embryos in CCR carriers undergoing PGT-SR.

### Cytogenetic analysis

Using the standard G-banding technique, cytogenetic analysis was performed on cultured peripheral blood lymphocytes from the 15 couples.

### Controlled ovarian stimulation and PGT-SR procedure

The procedures reported previously were followed (Liu et al., 2021; Dong et al., 2023). Briefly, controlled ovarian stimulation was induced using a gonadotropin-releasing hormone (GnRH) agonist, recombinant follicular stimulating hormone (FSH) and human chorionic gonadotropin (HCG). Standard techniques were employed in IVF treatment process at the Reproductive Medical Centre of Guangdong Women and Children Hospital, including fertilization, embryo culture, blastocyst biopsy, and blastocyst transfer. Blastocysts on day 5/6 with a grading score ≥3BC were selected for biopsy.

Trophectoderm (TE) cells obtained from day 5/6 biopsies for PGT-SR were subjected to whole-genome amplification (WGA) using the PicoPLEX single-cell WGA kit (Rubicon Genomics, Ann Arbor, United States). Subsequently, sequencing libraries were prepared using the WGA products of the embryos and then analyzed for the detection of copy number variation (CNV) in all 24 chromosomes using next-generation sequencing (NGS) following standard protocols. Euploid or mosaic embryos (Leigh et al., 2022), accompanied by genetic counseling, could be transferred into the uterine cavity.

### Statistical methods

The demographic characteristics and clinical outcomes were typically presented as mean values with standard deviations (SD) for continuous variables, and as frequency with proportion for categorical variables. The differences between groups were assessed using the ANOVA test for continuous variables and the Pearson’s chi-square test for categorical variables.

As multiple embryos from the same woman were included in the cohort, a multivariate generalized estimating equation (GEE) with an exchangeable working correlation matrix was utilized to examine the associations between patient demographics and embryonic euploidy. The following potential influencing factors were considered for inclusion in the GEE: female age (<35 years or ≥35 years), time of biopsy (day 3 or day 5/6), type of CCR (type A, B or C) and carrier’s sex (female or male). All statistical analyses were performed using R Version 4.3.1. All *p* values were two-sided, and less than 0.05 was considered statistically significant.

## Results

### Karyotyping

Fifteen couples, in which one partner was identified as a CCR carrier through G-banding analysis of peripheral blood (Supplementary Figure S1), were categorized into three groups: two couples belonged to type A, five to type B, and eight to type C (Table 1).

### Clinical characteristics and PGT-SR outcomes of CCR carriers

No statistically significant differences were observed among the three groups in terms of baseline information (Table 2).

**TABLE 2:** Demographic and embryologic characteristics of 15 CCR carriers.

	Type A	Type B	Type C	*p*-value
Demographic characteristic				
Female age (years)	31.00 ± 5.66	28.20 ± 3.03	29.63 ± 5.04	0.741
Male age (years)	31.00 ± 5.66	28.40 ± 2.30	32.25 ± 6.52	0.480
BMI (kg/m^2^)	21.90 ± 5.66	20.66 ± 1.24	22.37 ± 3.67	0.673
AMH (ng/mL)	8.05 ± 5.53	5.38 ± 0.69	5.59 ± 3.06	0.518
Embryologic characteristic				
2PN rate	74.5% (35/47)	79.2% (103/130)	74.8% (92/123)	0.655
Biopsied blastocyst rate	59.4% (19/32)	40.0% (40/100)	44.6% (41/92)	0.159
Euploid blastocyst rate	26.3% (5/19)	10% (4/40)	17.1% (7/41)	0.271

AMH, anti-Müllerian hormone; BMI, body mass index.

As presented in Tables 1, 2, 17 PGT-SR cycles were performed in 15 cases, resulting in the retrieval of 337 oocytes, with 300 (89.0%) available for fertilization. Subsequently, 230 (76.7%) oocytes developed into two-pronuclear embryos (2PN), and out of these, 224 (97.4%) embryos reached day 3 of development. Finally, a total of 100 (44.6%) blastocysts were eligible for biopsy and detection on day 5/6. The rates of 2PN in types A, B and C were found to be 74.5% (35/47), 79.2% (103/130) and 74.8% (92/123), respectively; however, no significant differences were observed among the three types (χ^2^ = 0.85, *p* = 0.655) (Table 2). Similarly, there were no significant differences in the rates of blastocyst formation on day 5/6 for biopsy between the three groups: 59.4% (19/32, type A), 40.0% (40/100, type B), and 44.6% (41/92, type C) respectively (χ^2^ = 3.68, *p* = 0.159) (Table 2). However, two patients (case 14 and 15) had no eligible blastocysts for biopsy within one PGT-SR cycle.

The NGS-based PGT-SR results are presented in Supplementary Table S1. All of the 100 biopsied blastocysts were diagnosed successfully, with 16.0% (16/100) embryos identified as balanced or normal, 79.0% (79/100) as aneuploid, and 5.0% (5/100) as mosaic (Table 1). In addition, 26 out of 100 embryos involved *de novo* abnormal chromosomes that were not present in the carriers. The rate of euploid blastocysts was found to be the highest in type A CCR at 26.3% (5/19), followed by type C with a rate of 17.1% (7/41). In contrast, type B CCR had the lowest rate at 10.0% (4/40). However, no statistically significant differences were observed among the three types (χ^2^ = 2.61, *p* = 0.271) (Table 2).

In the 17 PGT cycles, there were eight cycles (8/17, 47.1%) in which no euploid embryo could be transplanted. A total of 11 normal/balanced embryos (46, XN) (XN means XX or XY) and one mosaic embryo [46, XN, + (mosaic)(16)(q22.2-q24.3)(17.51 Mb)(40%)] (case 10, Supplementary Table S1) were transplanted with frozen-thawed embryo transfer, resulting in eight live births for 7 couples (cases 2, 5, 7, 9, 10, 12 and 13) (Table 1).

### Factors influencing the proportion of normal/balanced embryos

**FIGURE 1:**
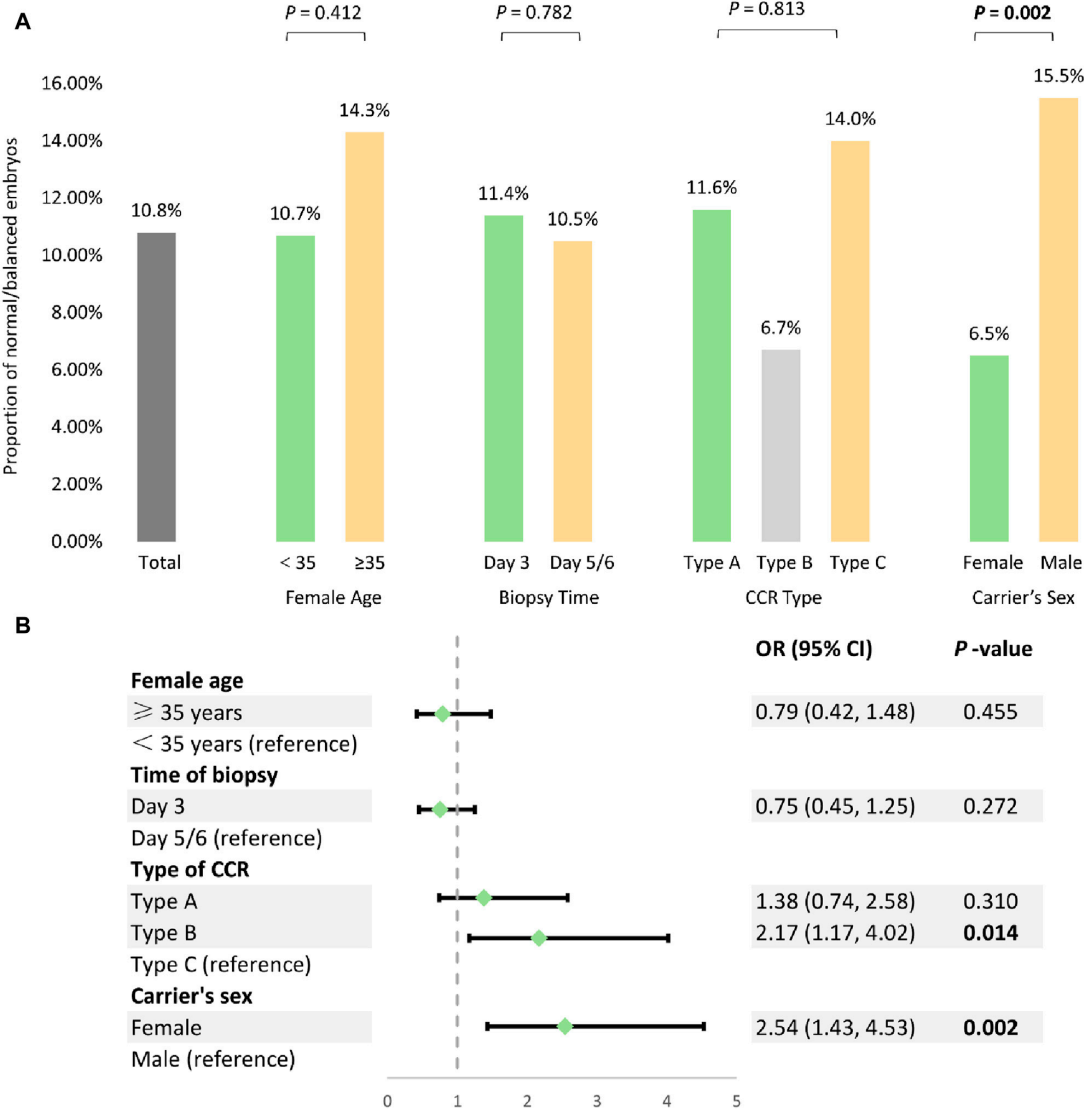
Analysis of the potential factors influencing PGT-SR outcomes among 54 CCR carriers. **(A)** Univariate analysis of the potential factors affecting normal/balanced embryo proportions. **(B)** The multivariable generalized estimating equations analysis to model predictors for normal/balanced embryos.

To date, an extensive literature review has identified a total of 39 individuals with balanced CCR who have undergone PGT-SR (Table 3). A total of 352 embryos were successfully tested on either day 3 or day 5/6. Consequently, 54 CCR carriers (24 females and 30 males), comprising 25 type A, 11 type B, and 18 type C cases, were included in the analysis. In summary, as presented in Figure 1A, the overall proportion of normal/balanced embryos among CCR carriers was 10.8% (49/452). The rates of euploid embryos for female aged <35 and ≥35 were 10.7% (39/363) and 14.3% (9/63), respectively (χ^2^ = 0.67, *p* = 0.412). Similarly, the euploid embryos rates for day 3 and day 5/6 biopsies were found to be 11.4% (18/158) and 10.5% (31/294), respectively (χ^2^ = 0.08, *p* = 0.782). Additionally, type B CCR exhibited the lowest euploid embryos rate at 6.7% (8/120), whereas type A and C displayed the rates of 11.6% (26/225) and 14.0% (15/107), respectively; however, the observed differences did not reach statistical significance (χ^2^ = 3.40, *p* = 0.183). Notably, the proportion of normal/balanced embryos was significantly lower in female carriers (6.5%, 15/232) compared to male heterozygotes (15.5%, 34/220) (χ^2^ = 9.44, *p* = 0.002).

**TABLE 3:** Summary of PGT-SR in previously reported patients carrying CCR.

References	Karyotype of CCR case	Female age (years)	CCR type	Biopsy time	Diagnosd embryos	Euploid embryos
Ren et al. (2023)	45, XY, t (3; 11) (q28; p15.4), der (14; 15) (q10; q10)	NA	B	Day 5/6	1	0
Özer et al. (2022)	46, XX, t (2; 6;12) (p21; p25; p13)	NA	A	Day 3	7	1
				Day 5/6	18	0
Dufton and Bouzayen (2021)	46, XX, t (5; 17) (p15.1; q25.1), ins (6; 17) (p23; q23q24)	35	C	Day 5/6	2	0
Ou et al. (2020)	46, XX, der (1)t (1; 4) (p22; q31.1), der (4)ins (5; 4) (q22; q25q28)t (1; 4),der (5)ins (5; 4)	27	C	Day 5/6	11	1
Li et al. (2020)	46, XY, t (1; 16; 4) (p22; q22; q23)	33	A	Day 5/6	2	1
	46, XY, t (8; 10; 13) (q21; p12; q33)	25	A	Day 5/6	7	2
	46, XX, t (1; 15; 9) (q21; q11.2; q12)	28	A	Day 5/6	4	0
	46, XY, t (8; 18; 9) (q24.2; q21.2; p22)	26	A	Day 5/6	15	0
	46, XY, t (2; 4) (q21; q31), t (2; 5) (p23; q35)	37	B	Day 3	5	1
	45, XX, t (6; 13) (p21.1; q34),der (15; 21) (q10; q10)	23	B	Day 5/6	13	0
	46, XX, t (1; 11) (q44; q23), t (2; 8) (q31; p23)	22	B	Day 5/6	8	0
	46, XX, t (2; 11) (q22; q24), inv (13) (q12q32)	37	C	Day 5/6	7	1
	46,XY, t (1; 11) (p10; p10), inv (11) (q13q14)	37	C	Day 5/6	6	0
	46, XY, t (1; 8) (p22; p23), ins (1; 11) (p22; q23q25)	24	C	Day 5/6	4	0
	45, XY, inv (1) (p11q12), der (15; 22) (q10; q10)	26	C	Day 5/6	1	0
	45, XY, inv (5) (p13q23),der (14; 15) (q10; q10)	32	C	Day 5/6	5	2
Mas et al. (2018)	46, XY, t (2; 4;14) (q21.1; p15.2; q22)	30	A	Day 5/6	10	0
Hu et al. (2018)	46, XX, t (2; 3;4) (p13; q13.2; q21)	<35	A	Day 5/6	2	0
	46, XX, t (1; 6;3) (p22; q21; p24)	<35	A	Day 5/6	2	0
	46, XX, t (2; 12; 4) (p13; p11; q33)	<35	A	Day 5/6	6	1
	46, XX, t (1; 12; 21) (q25; q15; q11)	<35	A	Day 5/6	8	1
	46, XY, t (2; 13; 9) (p23; q14; p11)	<35	A	Day 5/6	8	0
	46, XY, t (9; 16; 12) (p22; q22; q15)	<35	A	Day 5/6	8	1
	46, XX, t (6; 8) (q15; q24), t (1; 9;15) (q42; p11; q11q26)	<35	C	Day 5/6	12	0
Brunet et al. (2018)	46, XY, t (1; 4;11) (p31; p16; p22)	27	A	Day 5/6	3	1
	46, XY, t (3; 13; 5) (p14; q21; p14)	27	A	Day 5/6	9	0
	46, XX, t (6; 11; 21) (q21; q21; q13)	25	A	Day 5/6	6	2
	46, XX, t (2; 7) (q21; q36),t (2; 4) (p10; q10), t (2; 4) (q15; q10)	27	C	Day 5/6	2	0
Frumkin et al. (2017)	46, XY, t (3; 7;9) (q23; q22; q22)	29	A	Day 5/6	14	2
Scriven et al. (2014)	46, XY, t (6; 11; 16) (q16.2; p14.2; q13)	38	A	Day 3	5	0
	46, XY, t (1; 4;14) (p32.3; q23; q13)	30	A	Day 3	9	3
	46, XY, t (1; 9;18) (p13.3; p22; q23)	38	A	Day 3	15	3
	46, XX, t (1; 3;4) (q42.1; q26.2; p15.2)	33	A	Day 3	8	0
Vanneste et al. (2011)	46, XY, ins (3; 2) (p23; q23q14.2), t (6; 14) (p12.2; q13)	27	C	Day 3	16	4
Lim et al. (2008)	46, XX, t (6; 10; 8) (q25.1; q21.1; q21.1)	33	A	Day 3	11	0
	46, XY, t (5; 13; 8) (q21.2; q14.3; q24.3)	33	A	Day 3	7	1
Escudero et al. (2008)	45, XX, t (8; 12) (q24.1; q22), der (13; 14) (q10; q10)	33	B	Day 3	42	2
	45, XX, t (3; 9) (q10; p10), der (14; 15) (q10; q10)	35	B	Day 3	11	1
	46, XX, t (5; 13; 16) (q35.1; q32.1; q11.1)	33	A	Day 3	22	2

These potential factors were further analyzed using multivariate GEE to determine their impact on the proportion of balanced embryos following PGT-SR. The result indicated that type B CCR (OR = 2.17, 95% CI 1.17−4.02, *p* = 0.014) and female carrier (OR = 2.54, 95% CI 1.43−4.53, *p* = 0.002) were independent risk factors associated with a decrease in the proportion of euploid embryos, while female age (OR = 0.79, 95% CI 0.42−1.48, *p* = 0.455) and time of biopsy (OR = 0.75, 95% CI 0.45−1.25, *p* = 0.272) had no significant effect (Figure 1B).

## Discussion

Although CCR are uncommon events in humans, the frequency of CCR carriers in the PGT-SR population is described here for the first time. In our reproductive center, 1.9% (15/793) of couples undergoing PGT-SR cycles were identified as CCR carriers, who have normal physical health but may be at risk for spontaneous abortion or chromosomally abnormal offspring. The proportion of carriers with balanced CCR in the PGT-SR population has not been reported previously, and this proportion may exceed expectations.

We assessed the PGT-SR outcomes in 15 couples carrying three different types of CCR. Our data showed that the fertilization rates (76.7%), the embryo formation rates on day 3 (97.4%), and the blastocyst formation rates for biopsy (44.6%) were within the normal range, with no significant differences observed among the three groups (Table 2). Two studies have suggested some CCR may lead to poor early embryonic development (Hu et al., 2018; Li et al., 2020), which is consistent with our observed cases 3, 14 and 15. However, the phenomenon should be further investigated with larger samples to derive a more precise conclusion. Among the 100 blastocysts biopsied from 15 CCR couples after 17 PGT-SR cycles, only 16.0% were diagnosed as normal/balanced. The live birth rate after transfer of euploid embryos was 63.6% (7/11), and it was 100% (1/1) for mosaic embryo. Therefore, considering the relatively low incidence of normal/balanced embryos for CCR patients after PGT-SR cycles, transferring an embryo with a trophectoderm mosaic-range result could be considered as a viable clinical strategy (ASRM, 2023).

Several studies attribute a portion of the *de novo* aneuploidies to the inter chromosomal effect (ICE) (Lejeune, 1963; Wang et al., 2019), but the existence of ICE in CCR carriers remains controversial (Pellestor et al., 2011b; Wang et al., 2015; Ogur et al., 2023). In the present study, 26 out of 100 embryos involved unrelated chromosomal imbalances, providing limited evidence for the occurrence of ICE in CCR carriers. Comprehensive analysis (such as NGS) of all chromosomes in parallel with rearrangement-related chromosome testing is nonetheless essential.

In addition, for the first time, we conducted a systematic analysis using a relatively large sample size from our research and the published literature (Table 3). The results revealed that the odds of obtaining a euploid embryo was 10.8%, which was significantly lower compared to the genetically transferable embryos (26.8%, 3991/14883) in individuals with common chromosomal rearrangements (Coonen et al., 2020). After the GEE analysis of 452 embryos from 52 CCR individuals undergoing PGT-SR, we found that CCR type (OR = 2.17, 95% CI 1.17−4.02, *p* = 0.014) and carrier’s sex (OR = 2.54, 95% CI 1.43−4.53, *p* = 0.002) were independent risk factors that may be associated with the proportion of normal/balanced embryos (Figure 1B). Type B (double two-way translocations) CCR reduced the percentage of normal/balanced embryos, whereas a previous small-sample study demonstrated that different types of CCR had little effect on the embryonic molecular karyotype (Li et al., 2020). Also, the likelihood of obtaining at least one embryo for transfer following PGT-SR may be substantially less for female carriers, suggesting the different mechanisms and checkpoints involved in male and female meiosis (Zhang et al., 2019; Lin et al., 2021). Interestingly, the majority of familial CCR are transmitted through female carriers (70% maternal versus 30% paternal) (Pellestor et al., 2011a). This observation is mainly due to spermatogenesis failure in half of males, frequently linked with CCR and leading to sterility or subfertility (Liang et al., 2022). Additionally, some studies demonstrated that female age (Dang et al., 2023) and biopsy time (Beyer and Willats, 2017) might impact the proportion of genetically normal/balanced embryos for translocation carriers. Nevertheless, no significant influence of female age or biopsy time was observed on the normal/balanced embryos in our study. The possible reasons for this discrepancy could be the small sample size or the heterogeneity of CCR. However, larger cohort studies will be required to accurately assess the clinical outcomes, influencing factors, and efficacy of PGT-SR in carriers with CCR.

In conclusion, we evaluated the clinical outcomes of NGS-based PGT-SR in 15 carriers with three different types of CCR. This is the most extensive series of CCR carriers undergoing PGT-SR ever reported. PGT-SR may improve the reproductive outcomes in individuals with CCR, even though the proportion of normal/balanced embryos is relatively low. Moreover, type B CCR and female carrier are independent risk factors that may reduce the proportion of normal/balanced embryos. These findings may help optimize the genetic counseling and clinical management of these complex cases.

## Data Availability

The original contributions presented in the study are included in the article/Supplementary Material, further inquiries can be directed to the corresponding authors.
