# Supplementary material for: Preimplantation genetic testing for complex chromosomal rearrangements: clinical outcomes and potential risk factors
Source: Front Genet. 2024 Jul 29;15:1401549. doi: 10.3389/fgene.2024.1401549 (PMC11320417; doi:10.3389/fgene.2024.1401549)
Supplement: Supplementary file 1 [file Table1.PDF]

Supplementary Table S1. Next-generation sequencing (NGS) outcome of 15 CCR cases.

Case No.	Type	PGT-SR cycle	Biopsied Embryo	Embryo No.	Embryo score	NGS outcome	Diagnosis
1	A	1	3	1	6BB	46,XN	Euploid
				2	4BC	46,XN,+(2)(pter-p25.1)(12Mb),-(22)(q11.1-q13.32)(33Mb)	Aneuploid
				3	5BC	45,XN,-(22q)(q11.1-q13.32)(33Mb)	Aneuploid
2	A	1	16	1	4BB	46,XN,-(11)(q21-q25)(37.91Mb),+(14)(q31.1-q32.33)(24.09Mb)	Aneuploid
				2	4BB	46,XN,-(mos)(2)(q23.3-q37.3)(92.11Mb)(36%),+(mos)(7)(33%),+(mos)(11)(q12.1-q25)(79.05Mb)(30%),+(mos)(20)(39%)	Mosaic
				3	4BB	46,XN,+(mos)(1)(p31.1-p21.1)(22.29Mb)(32%),+(mos)(3)(30%),-5	Aneuploid
				4	4BB	46,XN,-(11)(q21-q25)(37.91Mb),+(14)(q31.1-q32.33)(24.09Mb)	Aneuploid
				5	4BB	45,XN,-(7)(q11.22-q36.3)(87.56Mb),-(11)(q21-q25)(37.91Mb),+(14)(q31.1-q32.33)(24.09Mb),-21	Aneuploid
				6	4BB	46,XN,+(5)(q23.2-q35.3)(58.62Mb),-(14)(q31.1-q32.33)(24.09Mb)	Aneuploid
				7	4BB	46,XN	Euploid
				8	4BB	46,XN	Euploid
				9	4BB	46,XN,+(11)(p15.5-q22.1)(97.58Mb),+(14)(q31.1-q32.33)(24.09Mb)	Aneuploid
				10	4BB	46,XN,+(11)(q21-q25)(37.91Mb),-(14)(q31.1-q32.33)(24.09Mb)	Aneuploid
				11	4BB	46,XN,+(5)(q23.2-q35.3)(58.62Mb),-(11)(q21-q25)(37.91Mb)	Aneuploid
				12	4BB	46,XN	Euploid
				13	4BB	46,XN,-(5)(q23.2-q35.3)(58.62Mb),+(11)(q21-q25)(37.91Mb)	Aneuploid
				14	4BC	46,XN	Euploid
				15	4BC	46,XN,+(5)(q23.2-q35.3)(58.62Mb),-(11)(q21-q25)(37.91Mb)	Aneuploid
				16	4BC	46,XN,-(11)(q21-q25)(37.91Mb),+(14)(q31.1-q32.33)(24.09Mb)	Aneuploid
3	B	1	2	1	4BC	46,XN,+(1)(q32.2-q44)(38Mb),-(14)(q24.1-q32.33)(37Mb)	Aneuploid
				2	4BC	46,XN,+(1)(q32.2-q44)(38Mb),-(mos)(6)(41%),-(14)(q24.1-q32.33)(37Mb)	Aneuploid
4	B	1	9	1	4BB	47,XN,+16	Aneuploid

				2	4BB	46,XN,+(3)(q23-q29)(56.74Mb),+(mos)(10)(q24.2-q26.3)(35.18Mb)(32%),-(22)(q12.1-q13.33)(25.24Mb)	Aneuploid
				3	4BC	45,X,-(3)(q23-q29)(58.54Mb),-(22)(q11.21-q12.1)(7.96Mb)	Aneuploid
				4	4BC	45,XN,-22	Aneuploid
				5	4BC	46,XN,+(4)(p15.33-q35.2)(178.12Mb),-(22)(q11.21-q12.1)(7.96Mb)	Aneuploid
				6	4BC	45,XN,-22	Aneuploid
				7	4BC	46,XN,-(mos)(2)(49%),+(4)(p16.3-p15.33)(12.31Mb),-(4)(p15.33-q35.2)(178.12Mb),-(22)(q12.1-q13.33)(25.24Mb)	Aneuploid
				8	4BC	46,XN,+(4)(p15.33-q35.2)(178.12Mb),+(22)(q12.1-q13.33)(25.24Mb)	Aneuploid
				9	4BC	46,XN,-(4)(p15.33-q35.2)(178.12Mb),+(22)(q11.21-q12.1)(7.96Mb)	Aneuploid
5	B	1	12	1	4BB	46,XN,+(3)(q26.33-q29)(18Mb),-(13)(q21.33-q34)(41Mb)	Aneuploid
				2	4BB	46,XN,(mos)(-1)(p36.22-p36.12)(14Mb)(52%),+(3)(q26.33-q29)(17Mb),(mos)(-7)(q22.1-q36.3)(57Mb)(46%),-(mos)(9)(51%),-(mos)(12)(q21.2-q23.1)(24Mb)(63%),-(13)(q21.33-q34)(41Mb)	Aneuploid
				3	4BB	46,XN,-(3)(q26.33-q29)(17Mb),-(13)(q12.11-q21.33)(53Mb)	Aneuploid
				4	4BB	46,XN	Euploid
				5	6BB	46,XN	Euploid
				6	5CB	46,XN,+(mos)(5)(30%),-(mos)(7)(q11.21-q11.22)(11Mb)(30%)	Mosaic
				7	6BB	46,XN,-(3)(q26.33-q29)(17Mb),+(13)(q21.33-q34)(41Mb)	Aneuploid
				8	4BB	46,XN,-(3)(q26.33-q29)(17Mb),+(13)(q21.33-q34)(41Mb)	Aneuploid
				9	4BB	46,XN,+(3)(p26.3-p11.1)(89Mb),+(3)(q11.2-q29)(103Mb),-5	Aneuploid
				10	4BC	46,XN,+(3)(p26.3-p11.1)(89Mb),+(3)(q11.2-q29)(103Mb),+(6)(q25.3-q27)(10Mb)(56%),+(14)(q11.1-q32.33)(88Mb)	Aneuploid
				11	4BC	46,XN,+(3)(p26.3-p11.1)(89Mb),+(3)(q11.2-q26.33)(86Mb),-(3)(q26.33-q29)(17Mb),-(13)(q12.11-q21.33)(53Mb),+(mos)(13)(q12.11-q21.33)(53Mb)(62%)	Aneuploid
				12	4BC	46,XN,-(3)(q26.33-q29)(17Mb),-(13)(q12.11-q21.33)(53Mb)	Aneuploid
6	B	1	6	1	4BB	46,XN,+(2)(mos)(q14.2-q37.3)(122.60Mb)(52%),-(5)(q14.3q35.3)(96.32Mb),+(6)(q22.33q27)(41.72Mb)	Aneuploid
				2	4BC	46,XN,+1,+(5)(q14.3-q35.3)(96.32Mb),-(6)(q22.33-q27)(41.52Mb),-7	Aneuploid

				3	4BA	46,XN,-(5)(p15.33-q14.3)(85.00Mb),-(6)(p25.3-q22.33)(129.80Mb),+(14)(mos)(q11.2-q31.3)(68.00Mb)(38%)	Aneuploid
				4	4BC	46,XN,-(8)(mos)(62%)	Mosaic
				5	4BC	45,XN,-7	Aneuploid
				6	4BC	46,XN,+1,+(5)(q14.3-q35.3)(96.32Mb),-(6)(q22.33-q27)(41.52Mb),-7	Aneuploid
7	B	1	11	1	4BB	46,XN,-(15)(q25.1-q26.3)(21.71Mb)	Aneuploid
				2	4AA	46,XN,-(15)(q25.1-q26.3)(21.71Mb)	Aneuploid
				3	4AA	46,XN	Euploid
				4	6BB	46,XN,-(15)(q25.1-q26.3)(21.71Mb)	Aneuploid
				5	6BB	46,XN	Euploid
				6	5BC	46,XN,+(4)(p16.3-q28.1)(127.64Mb),-(5)(p15.33-q32)(147.23Mb)	Aneuploid
				7	4BB	46,XN,+(4)(q28.1-q35.2)(62.79Mb),-(5)(q32-q35.3)(33.67Mb),+(15)(q25.1-q26.3)(21.71Mb)	Aneuploid
				8	4BB	46,XN,-(15)(q25.1-q26.3)(21.71Mb)	Aneuploid
				9	4BB	46,XN,-(15)(q25.1-q26.3)(21.71Mb)	Aneuploid
				10	4BB	46,XN,+(4)(p16.3-q28.1)(127.64Mb),-(5)(p15.33-q32)(147.23Mb)	Aneuploid
				11	4BB	46,XN,-(15)(q25.1-q26.3)(21.71Mb)	Aneuploid
8	C	1	7	1	4AB	46,XN	Euploid
				2	4AB	46,XN,+(7)(q36.1-q36.3)(9Mb),-(22)(q12.3-q13.32)(13Mb)	Aneuploid
				3	4AB	46,XN,-(7)(q36.1-q36.3)(9Mb),+(22)(q12.3-q13.32)(13Mb)	Aneuploid
				4	4AB	46,XN,+(7)(q36.1-q36.3)(9Mb),-(22)(q12.3-q13.32)(13Mb)	Aneuploid
				5	4BB	46,XN,-(21)(q11.2-q22.2)(27Mb)	Aneuploid
				6	4BB	46,XN,-(7)(q36.1-q36.3)(9Mb),+(mos)(9)(54%),+(22)(q12.3-q13.32)(13Mb)	Aneuploid
				7	4BC	46,XN,-(7)(p22.3-q36.1)(148Mb),+7(q36.1-q36.3)(9Mb),+(mos)(22)(q11.1-q12.3)(20Mb)(60%),-(22)(q12.3-q13.32)(13Mb),-(22)(q13.33-qter)(1.3Mb)	Aneuploid
9	C	1	3	1	4BC	46,XN	Euploid
				2	4BC	46,XN,-(7)(p22.3-p12.3)(46.80Mb),+(8)(q12.1-q24.3)(87.37Mb)	Aneuploid

				3	5BB	46,XN,-(7)(p12.3-q36.3)(111.45Mb),+(8)(p23.3-q12.1)(58.93Mb)	Aneuploid
		2	6	1	5BC	46,XN,+(mos)(1)(41%),+(4)(q23-q35.2)(92.19Mb)	Aneuploid
				2	4BB	47,XN,+(7)(p22.3-p12.3)(47.40Mb),+(8)(q11.21-q24.3)(98.17Mb),+12	Aneuploid
				3	4BB	46,XN,-(16)(13.3 13.12)(14 03Mb),+(mos)(16)(p13.12-q24.3)(72.01Mb) (65%)	Aneuploid
				4	3BB	47,XN,+7	Aneuploid
				5	4BC	45,XN,-4,+(7) (p22.3-p12.3)(47.40Mb),-(8)(q12.1-q24.3)(87.37Mb)	Aneuploid
				6	3BC	46,XN	Euploid
10	C	1	5	1	4AB	46,XN,-(mos)(1)(p36.33-p34.3)(35.51Mb)(50%),+(2)(p12-q37.3)(163.76Mb),-(18)(p11.32-q12.1)(32.30Mb)	Aneuploid
				2	4BC	46,XN, -(2)(p12-q37.3)(163.76Mb),+(18)(p11.32-q12.1)(32.30Mb)	Aneuploid
				3	4BC	47,XN,+(2)(p12-q37.3)(163.76Mb),-(mos)(11)(q14.2-q25)(47.77Mb)(69%),+16,-(18)(p11.32-q12.1)(32.30Mb)	Aneuploid
				4	4BC	46,XN,+(2)(p25.3-p12)(79.33Mb),-(18)(q12.1-q23)(45.26Mb)	Aneuploid
				5	4BC	46,XN,+(2)(p25.3-p12)(79.33Mb),-(18)(q12.1-q23)(45.26Mb)	Aneuploid
		2	3	1	4BC	46,XN,-(2)(p25.3-p12)(79.33Mb),+(18)(q12.1-q23)(45.70Mb)	Aneuploid
				2	5BC	46,XN,-(mos)(8)(q22.1-q24.3)(51.18Mb)(42%),-(9)(q21.33-q22.33)(11.64Mb)	Aneuploid
				3	5BC	46,XN,+(mos)(16)(q22.2-q24.3)(17.51Mb)(40%)	Mosaic
11	C	1	4	1	5BB	46,XN,+(4)(q33-q35.2)(19.59Mb),-(14)(q21.1-q32.33)(67.89Mb)	Aneuploid
				2	5BB	45,XN,-4,+(14)(q21.1-q21.3)(10.80Mb),+(14)(q24.2-q32.33)(34.29Mb),+(21)(q11.2-q21.3)(13.20Mb)	Aneuploid
				3	5BB	46,XN,+(14)(q24.2-q32.33)(34.29Mb),-(21)(q21.3-q22.3)(21.18Mb)	Aneuploid
				4	4BC	46,XN,+(mos)(4)(q32.3-q35.2)(22.59Mb),-(mos)(10)(42%),-(14)(q11.2-q21.1)(18.60Mb),-(14)(q22.1-q24.2)(20.40Mb)	Aneuploid
12	C	1	5	1	6BB	46,XN,-(4)(q34.1-q35.2)(15.39Mb),+(11)(q24.1-q25)(11.51Mb)	Aneuploid
				2	6BB	46,XN,+(4)(q34.1-q35.2)(15.39Mb),-(11)(q24.1-q25)(11.51Mb)	Aneuploid
				3	4BB	46,XN	Euploid
				4	4BB	46,XN,+(4)(q34.1-q35.2)(15.39Mb),-(11)(q24.1-q25)(11.51Mb),-(mos)(15)(q25.2-q26.3)(17.49Mb)(69%)	Aneuploid
				5	4BC	46, XN,-(4)(q34.1-q35.2)(15.39Mb),+(11)(q24.1-q25)(11.51Mb)	Aneuploid

13	C	1	8	1	4BC	46,XN,-(2)(q34)(31Mb),+(10)(q23.32)(42Mb)	Aneuploid
				2	6BC	46,XN	Euploid
				3	4BB	46,XN,-(2)(q34)(31Mb),+(10)(q23.32)(42Mb)	Aneuploid
				4	4BB	46,XN	Euploid
				5	4BB	46,XN	Euploid
				6	4BC	46,XN,-(mos)(2)(q24.1-q34)(56Mb)(60%),+(mos)(2)(q34)(27Mb)(40%),+(mos)(7)(q32.1-q34)(13Mb)(50%),-(mos)(8)(p23.1)(12Mb)(40%),-(10)(q23.32)(42Mb)	Aneuploid
				7	4BC	46,XN,-(2)(q34)(31Mb),+(mos)(9)(40%),+(mos)(10)(p14-p11.21)(30M)(60%),+(mos)(10)(q11.21-q23.31)(50Mb)(40%),-(10)(q23.32)(42Mb)	Aneuploid
				8	4BC	46,XN,+(mos)(12)(q24.31)(10.8M)(60%)	Mosaic
14	C	1	0	/	/	/	/
15	C	1	0	/	/	/	/

XN means XX or XY.

“mos” means mosaic.

“+” indicates the duplication of a whole chromosome or a segment of a chromosome.

“-” indicates the deletion of a whole chromosome or a segment of a chromosome.
