# Supplementary material for: Preimplantation genetic testing for complex chromosomal rearrangements: clinical outcomes and potential risk factors
Source: Front Genet. 2024 Jul 29;15:1401549. doi: 10.3389/fgene.2024.1401549 (PMC11320417; doi:10.3389/fgene.2024.1401549)

Supplementary Figure S1. G-banded karyotypes of 15 CCR carriers.

46,XX,t(2;9;22)(p25.2;q32;q11.1)

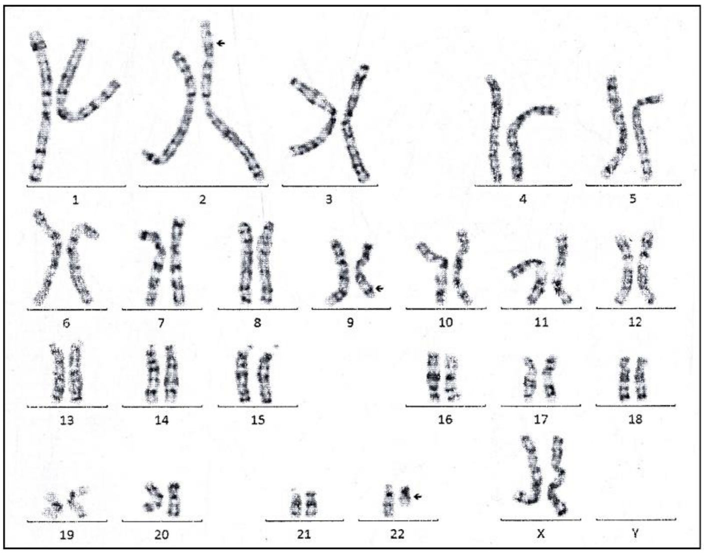

46,XY,t(5;14;11)(q23;q24.3;q21)

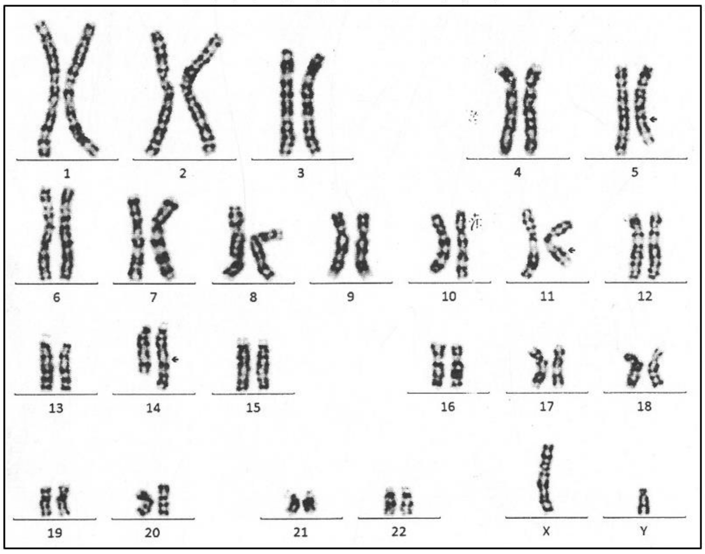

46,XY,t(1;14)(q32.3;q24.3),t(2;9)(p11.2;p24.2)

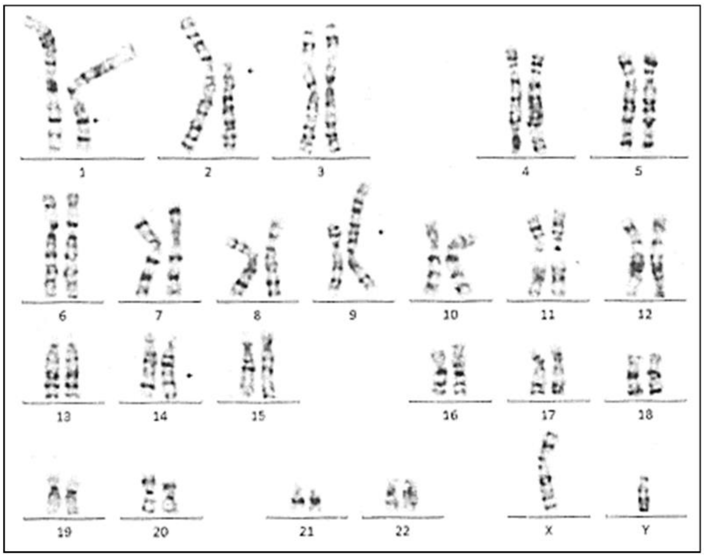

46,XY,t(3;4)(q21;p15.2)t(4;22)(p16.1;q12.2)

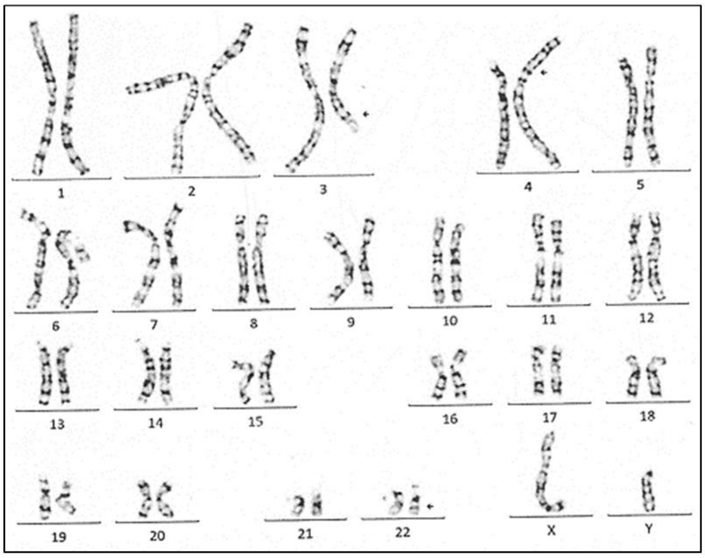

45,XY,t(3;13)(q26.2;q21.3),der(13;14)(q10;q10)

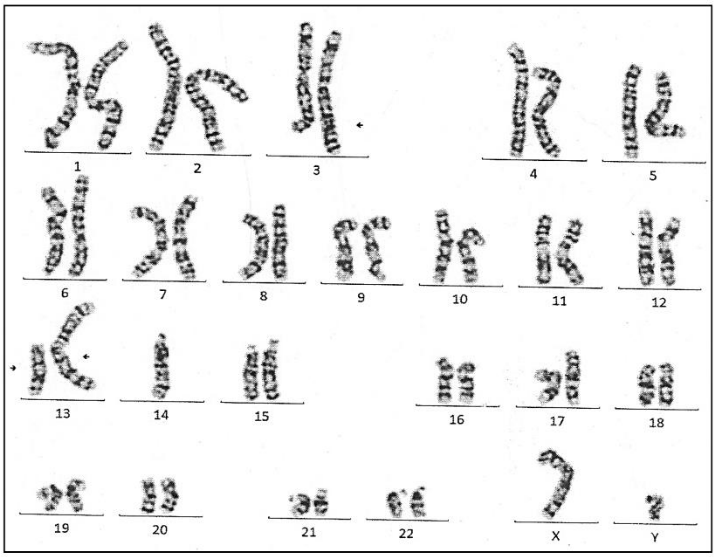

46,XX,t(1;7)(p13.1;q11.23),t(5;6)(q15;q23)

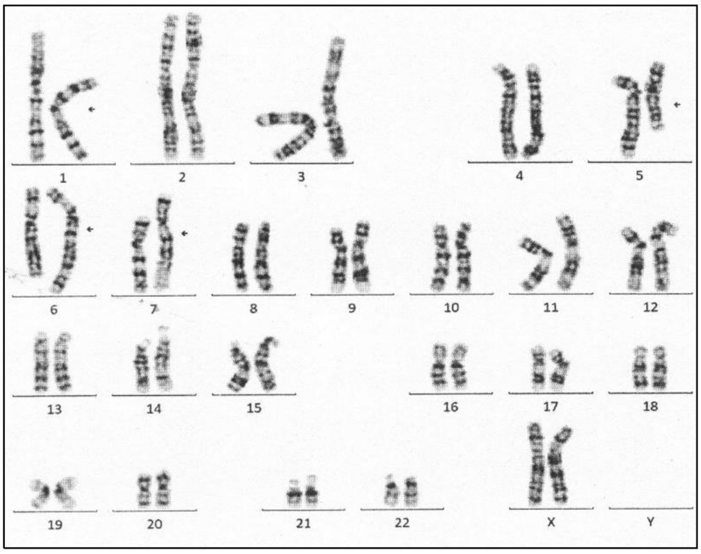

46,XY,t(4;5)(q27;q31),t(6;15)(q27;q25)

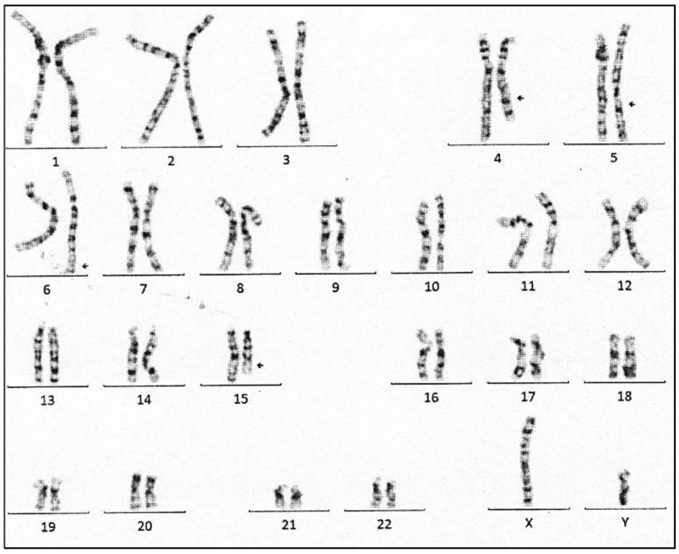

46,XY,t(7;22)(q36;q13.1)inv(22)(q12.2q13.1)

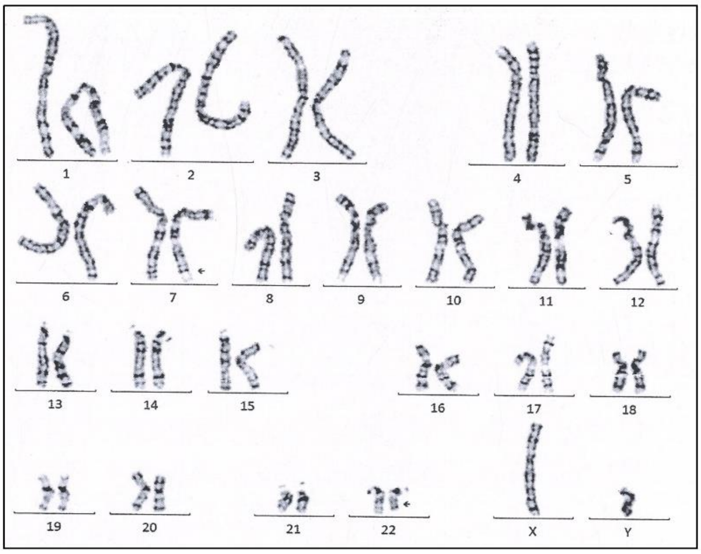

46,XX,t(7;8)(p13;q11.23)inv(7)(p13q21.2)

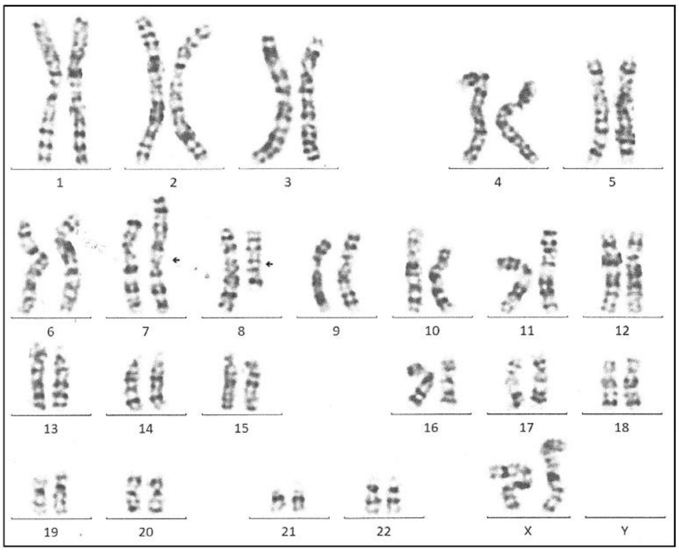

46,XX,t(2;18)(p10;p10),inv(9)(q21.2q22.3)

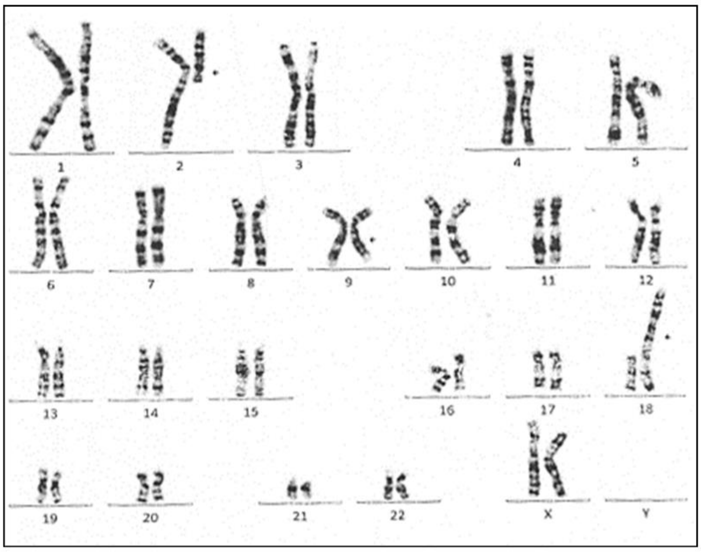

46,XX,der(4)ins(4;14)(q33;q22q24.2)t(4;21)(q34;q22.1),der(14)ins(14;21)(q11.2;q11.2q21)ins(21;14)(q22.1;q11.2q21)ins(4;14),der(21)ins(14;21)ins(21;14)t(4;21)

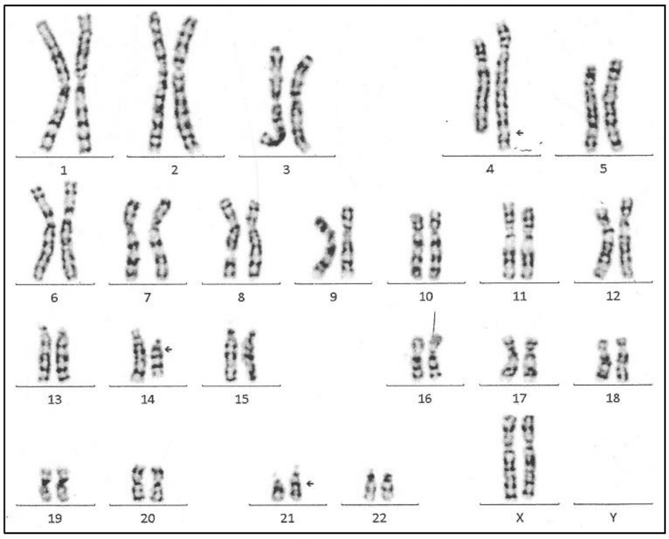

46,XY,der(4)inv(4)(q32q35)t(4;11)(q32;q25),der(11)t(4;11)

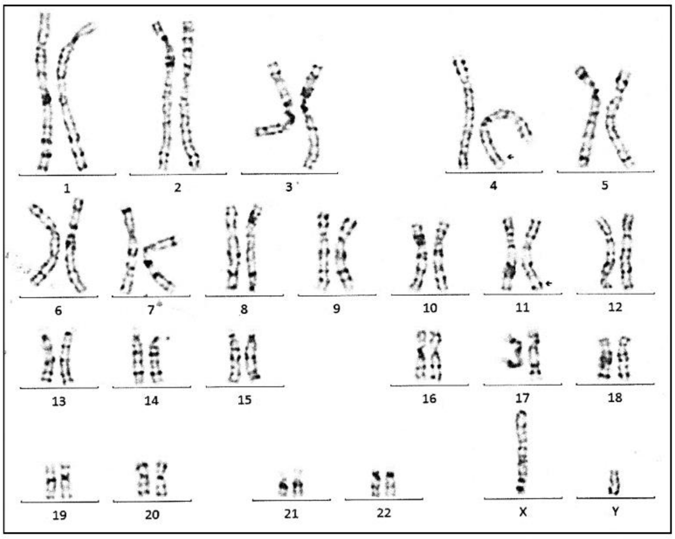

46,XY,der(2)t(2;10)(q35;q24.3),der(10)inv(10)(q22.1q24.3)t(2;10)

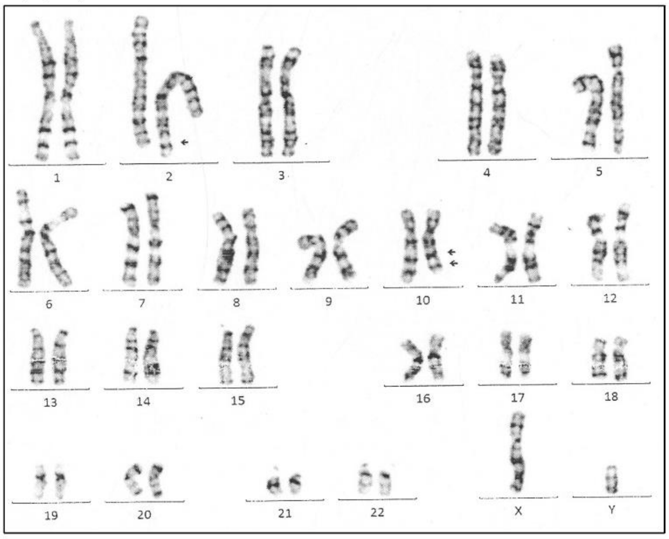

46,XY,der(5)ins(7;5)(p22;p15.1p15.3)t(5;6)(p15.1;q23),der(6)ins(7;6)(p22;q22q23)t(5;6),der(7)ins(7:5)ins(7:6)

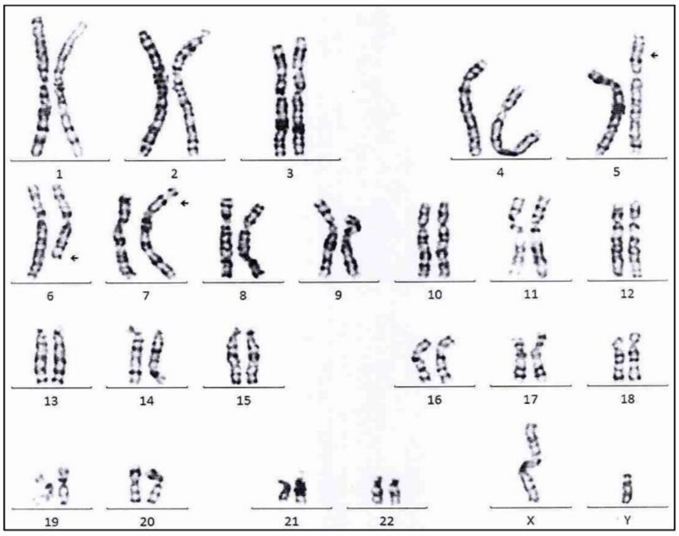

46,XY,der(3)t(3;9)(p22;q34.1),der(9)inv(9)(q33q34.3)t(3;9)

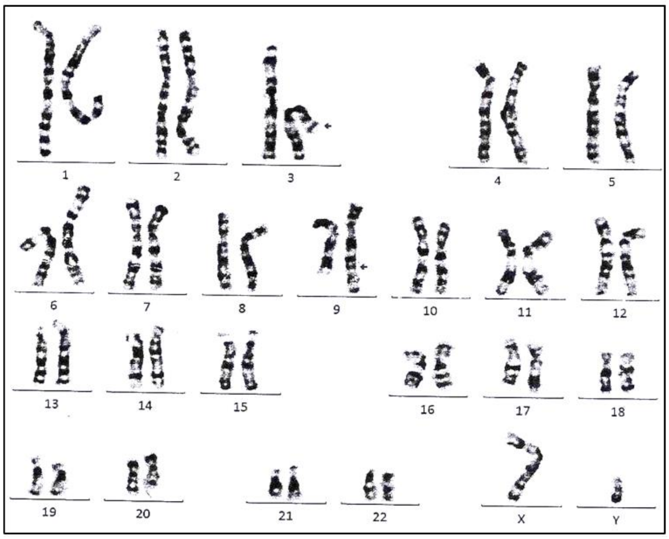

Supplement: Supplementary file 2 [file Image1.PDF]
